# Prenatal exposure to paracetamol is associated with smaller testicular volume: a COPANA cohort study of 287 healthy infant boys

**DOI:** 10.1093/hropen/hoag089

**Published:** 2026-10-06

**Authors:** Gylli Mola, Margit Bistrup Fischer, Hanne Frederiksen, Jørgen Holm Petersen, Lone Scheel, Katrine Bak Wraae, Ane Lilleøre Rom, Hanne Kristine Hegaard, David Møbjerg Kristensen, Anders Juul, Casper P Hagen

**Affiliations:** Department of Growth and Reproduction, Copenhagen University Hospital—Rigshospitalet, Copenhagen, Denmark; International Centre for Research and Research Training in Endocrine Disruption of Male Reproduction and Child Health (EDMaRC), Copenhagen University Hospital—Rigshospitalet, Copenhagen, Denmark; Department of Growth and Reproduction, Copenhagen University Hospital—Rigshospitalet, Copenhagen, Denmark; International Centre for Research and Research Training in Endocrine Disruption of Male Reproduction and Child Health (EDMaRC), Copenhagen University Hospital—Rigshospitalet, Copenhagen, Denmark; Department of Growth and Reproduction, Copenhagen University Hospital—Rigshospitalet, Copenhagen, Denmark; International Centre for Research and Research Training in Endocrine Disruption of Male Reproduction and Child Health (EDMaRC), Copenhagen University Hospital—Rigshospitalet, Copenhagen, Denmark; Department of Growth and Reproduction, Copenhagen University Hospital—Rigshospitalet, Copenhagen, Denmark; Center of Fetal Medicine and Pregnancy, Department of Obstetrics, Copenhagen University Hospital—Rigshospitalet, Copenhagen, Denmark; Center of Fetal Medicine and Pregnancy, Department of Obstetrics, Copenhagen University Hospital—Rigshospitalet, Copenhagen, Denmark; Department of Gynaecology, Fertility and Obstetrics, Copenhagen University Hospital—Rigshospitalet, Copenhagen, Denmark; Research Unit of Gynaecology and Obstetrics, Department of Clinical Research, University of Southern Denmark, Odense, Denmark; Department of Gynaecology, Fertility and Obstetrics, Copenhagen University Hospital—Rigshospitalet, Copenhagen, Denmark; Department of Clinical Medicine, University of Copenhagen, Copenhagen, Denmark; Department of Growth and Reproduction, Copenhagen University Hospital—Rigshospitalet, Copenhagen, Denmark; Department of Science and Environment, Roskilde University, Roskilde, Denmark; Department of Growth and Reproduction, Copenhagen University Hospital—Rigshospitalet, Copenhagen, Denmark; International Centre for Research and Research Training in Endocrine Disruption of Male Reproduction and Child Health (EDMaRC), Copenhagen University Hospital—Rigshospitalet, Copenhagen, Denmark; Department of Clinical Medicine, University of Copenhagen, Copenhagen, Denmark; Department of Growth and Reproduction, Copenhagen University Hospital—Rigshospitalet, Copenhagen, Denmark; International Centre for Research and Research Training in Endocrine Disruption of Male Reproduction and Child Health (EDMaRC), Copenhagen University Hospital—Rigshospitalet, Copenhagen, Denmark; Department of Clinical Medicine, University of Copenhagen, Copenhagen, Denmark

**Keywords:** paracetamol, pregnancy, prenatal exposure, testicular volume, minipuberty, male reproductive development

## Abstract

**STUDY QUESTION:**

Is prenatal exposure to paracetamol associated with testicular function in male offspring?

**SUMMARY ANSWER:**

Prenatal paracetamol exposure was associated with smaller testicular volume in infant boys.

**WHAT IS KNOWN ALREADY:**

Paracetamol (also known as acetaminophen) is available over the counter and is frequently used by pregnant women. Animal studies and secondary analyses of mother–child cohorts suggest paracetamol can adversely affect testicular development. To our knowledge, this is the first cohort study primarily designed to test the association between prenatal paracetamol exposure and postnatal testicular function.

**STUDY DESIGN, SIZE, DURATION:**

Prospective, observational pregnancy and birth cohort at a university hospital (2020–2022). Healthy, singleton pregnant women (n = 685) were enrolled during the first trimester, and 589 (287 boys) infants completed follow-up examination at 3 months of age.

**PARTICIPANTS/MATERIALS, SETTING, METHODS:**

Data on maternal paracetamol use (yes/no and dose) were collected prospectively every 2 weeks throughout pregnancy. Based on the timing of fetal masculinization, participants were classified as exposed at any time during fetal life (n = 137), early exposure (gestational week (GW) **≤** 15; n = 81), late exposure (GW **>**15; n = 118), or exposed during both early and late fetal life (n = 62). Unexposed boys served as controls (n = 150).

The boys were examined at infancy (i.e. minipuberty at mean age 3.1 months ±0.3 SD). Predefined primary outcomes: anogenital distance (AGD), testicular volume, and circulating levels of testosterone.

**MAIN RESULTS AND THE ROLE OF CHANCE:**

Across exposure windows, prenatal paracetamol exposure was consistently associated with smaller testicular volume at 3 months of age. Effect estimates were largest in boys exposed at early as well as late fetal life; dose of paracetamol, median (interquartile range (IQR)) 8.5 g (4.0–16.6), testicular volume (β) −34.1 mm³, 95% CI −65.5 to −2.7, *P* = 0.033.

**LIMITATIONS, REASONS FOR CAUTION:**

Residual confounding by indication cannot be excluded. The generalizability of the study may be affected by the homogeneity of the Copenhagen Analgesic Study (COPANA) cohort. Longitudinal follow-up is necessary for evaluation of reproductive health in adulthood.

**WIDER IMPLICATIONS OF THE FINDINGS:**

In this prospective human cohort study, prenatal paracetamol exposure was associated with reduced testicular volume during minipuberty, a transient period of postnatal activation of the hypothalamic–pituitary–gonadal axis that is considered a critical window for assessing future reproductive function.

**FUNDING:**

Rigshospitalet Research Council supports this work under grants (E-22778-02) and (E-22717-21). The research was furthermore funded by Læge Sofus Carl Emil Friis og hustru Doris Friis‘Legat (F-23936-01), Aase og Ejnar Danielsens Foundation (20-10-0367), Helsefonden (20-B-0388), Axel Muusfeldt fond (2020-0385), and The Danish Centre for Endocrine Disrupting Substances (CeHoS) (2022-23219). The publication was completed as part of the MERLON project under grant agreement No. 101137411. The MERLON project is funded by the European Union. Views and opinions expressed are, however, those of the authors only and do not necessarily reflect those of the European Union or the European Health and Digital Executive Agency (HADEA). Neither the European Union nor HADEA can be held responsible for them.

**DISCLOSURES:**

D.M.K. has received speaker fees from Merck. A.J. has received speaker fees from Ipsen, Novo Nordisk, and Sandoz. A.J. is Head of Department at a department that has received unrestricted research grants from Novo Nordisk and Lundbeck. All other authors declare no conflicts of interest.

**TRIAL REGISTRATION NUMBER:**

The Copenhagen Analgesic Study (COPANA) (ClinicalTrials.gov NCT04369222).

WHAT DOES THIS MEAN FOR PATIENTS? This study examined whether paracetamol use during pregnancy was associated with testicular development and function in infant boys.

Paracetamol is commonly used during pregnancy and is generally considered safe. However, experimental studies have raised concerns that exposure during fetal life may affect development of the male reproductive system. Compared with previous human studies, our prospective design included detailed information on paracetamol use throughout pregnancy and a thorough examination of parameters related to testicular development and function in infant boys.

Pregnant women reported their paracetamol use biweekly throughout pregnancy, and 287 infant boys were examined during infancy. Examinations included testicular volume by ultrasound, anogenital distance (a marker associated with fetal exposure to male sex hormones), and blood testosterone levels.

We found that prenatal paracetamol exposure was associated with smaller testicular volume in infant boys. Boys exposed during both early and late fetal life had, on average, 8% smaller testicular volume compared with unexposed boys.

Because this was an observational study, we cannot conclude that paracetamol caused the observed difference. However, the findings suggest that prenatal paracetamol exposure may be associated with aspects of testicular development and function. The long-term clinical importance remains unknown, and further studies are needed to follow exposed children over time to determine whether the observed differences persist and have implications for reproductive health later in life.

## Introduction

Male reproductive development is particularly vulnerable during early fetal life when testicular androgen production and action drive genital masculinization (Nistal *et al.*, 2015; Li *et al.*, 2023). Disturbed fetal testicular development during the masculinization programming window (MPW; gestational weeks 8–14) (Welsh *et al.*, 2008) has been proposed to contribute to a spectrum of male reproductive disorders, including hypospadias and cryptorchidism in infancy, as well as impaired semen quality and an increased risk of testicular cancer in adulthood (Skakkebaek *et al.*, 1987; Juul *et al.*, 2014), as described in the testicular dysgenesis syndrome (TDS) hypothesis (Skakkebaek *et al.*, 2001). Consistent with this framework, anogenital distance (AGD), a sensitive marker of fetal androgen exposure which can be evaluated already from the third trimester (Fischer *et al.*, 2024a), has been repeatedly associated with adverse male reproductive outcomes (Mendiola *et al.*, 2011; Jain and Singal, 2013; Cox *et al.*, 2017; Moreno-Mendoza *et al.*, 2020). Although the MPW is considered the critical period for androgen-dependent programming of the male reproductive tract, testicular development, and steroidogenesis continue throughout gestation (Nistal *et al.*, 2015), suggesting that prenatal exposure after the MPW also influences fetal testicular development and function.

Paracetamol (also known as acetaminophen) is used by ∼50% of pregnant women (Zafeiri *et al.*, 2021; Taagaard *et al.*, 2023) and readily crosses the placenta, leading to direct fetal exposure (Farre *et al.*, 2008; Nitsche *et al.*, 2017). Although generally considered safe, accumulating evidence from *in vivo* and *in vitro* studies, as well as secondary analyses of epidemiological data from established human birth cohorts, suggests that paracetamol may have endocrine-disrupting properties that can impair gonadal development (Bauer *et al.*, 2021). Epidemiological studies suggest that effects depend on both timing and dose of prenatal paracetamol exposure during fetal life (Kristensen *et al.*, 2011; Snijder *et al.*, 2012).

Paracetamol inhibits cyclooxygenase (COX) enzyme activity, and thereby reduces prostaglandin synthesis (Anderson, 2008; Mazaud-Guittot *et al.*, 2013). Prostaglandins play important roles in fetal testicular development, including the regulation of germ cell migration and proliferation, as well as differentiation of somatic cells within the developing gonad (Gupta and Goldman, 1986; Moniot *et al.*, 2014; Dean *et al.*, 2016). Reduced prostaglandin signaling has therefore been proposed as one mechanism by which paracetamol may disrupt normal fetal testicular development.

In addition, experimental studies suggest that prenatal paracetamol exposure may interfere with fetal steroidogenesis by decreasing expression of specific cytochrome P450 (CYP) enzymes during the MPW, potentially leading to impaired testosterone production (Kristensen *et al.*, 2012; Holm *et al.*, 2015; van den Driesche *et al.*, 2015).

The functional consequences of early perturbations in these pathways may become apparent during minipuberty, the transient activation of the hypothalamic–pituitary–gonadal (HPG) axis in infancy (Juul, 2026). During this period, gonadotropin stimulation drives processes critical for maturation of the testicular cellular architecture, including finalization of Sertoli cell numbers, initiation of germ cell differentiation, and coordination of somatic-germ cell signaling essential for future fertility (França *et al.*, 2016; Neto *et al.*, 2021; Sanou *et al.*, 2026). As such, minipuberty represents an early window of opportunity to assess postnatal gonadal function (Grumbach, 2005).

The Copenhagen Analgesic Study (COPANA) is a pregnancy and birth cohort specifically designed to test the hypothesis that fetal paracetamol exposure is associated with impaired testicular development and function, including primary outcomes reflecting general testicular development (testicular volume), fetal androgen signaling (assessed by AGD), and postnatal testicular function (circulating levels of testosterone) in 287 healthy infant boys.

## Materials and methods

### Ethics

The COPANA Study (ClinicalTrials.gov ID: NCT04369222) was approved by the Ethics Committee of the Capital Region of Denmark (H-19044036) and the Danish Data Protection Agency (P-2019-330). Written informed consent was obtained from all participants; for infant participants, consent was provided by all adults with legal guardianship.

### Population

COPANA is a single-centre, prospective, observational pregnancy, and birth cohort conducted at Copenhagen University Hospital—Rigshospitalet, Denmark, between 2020 and 2022. Details of the study protocol have been published previously (Fischer *et al.*, 2024b). As part of routine clinical care, all pregnant women completed an initial web-based clinical questionnaire (Q1) at approximately gestational week (GW) 10 when booking their first-trimester ultrasound scan (mean (SD) GW 9.9 (1.6)). Eligible pregnant women were identified by a midwife and invited, together with their partners, to participate in the study. Women with thyroid dysfunction or gestational diabetes were excluded. Inclusion criteria comprised Caucasian ethnicity and a pre-pregnancy BMI of 18–35 kg/m². After inclusion, women with multiple pregnancies, pre- or post-term delivery (before gestational week (GW) 37 or beyond GW 42), or serious infant illness were excluded. A total of 685 pregnant women (∼20% of 3425 eligible) consented to participate. After 96 participants were lost to follow-up, 589 families attended the child examination, including 287 boys.

### Questionnaires

Q1 captured current self-reported medication use and was routinely used in antenatal counseling and care. Participants were specifically asked: *‘Do you currently take any medication (including painkillers)?’* (translated from Danish). For mild analgesics, information was collected on consumption (yes/no; rarely/1–2 times weekly/daily) and drug name.

At enrolment (mean (SD) GW 14.2 (2.0)), the women completed an additional questionnaire on general and reproductive health (Q2). From enrolment onwards, women completed detailed electronic questionnaires every 2 weeks throughout pregnancy (Q3), reporting all medication use with a particular focus on mild analgesics, including both generic and brand names of all mild analgesic products available in Denmark. For each reported use, information was collected on consumption (yes/no), drug name, dose per use (mg), and indication. The first Q3 questionnaire covered the period from invitation to inclusion, providing continuous information on medication use from the first study contact (GW 9.9 (1.6)). The cumulative dose was calculated as the sum of all reported doses during pregnancy. After delivery, the questionnaire included questions regarding medication administered to the infant.

Participating families attended three study visits: during the first trimester (enrolment), during the third trimester for fetal ultrasound assessment, and at ∼3 months postpartum during the minipuberty for infant examination (Supplementary Fig. S1).

### Third-trimester ultrasound scan

A study-specific ultrasound examination was performed during the third trimester (GW 29–34) using a Voluson E10 system (GE HealthCare, Chicago, IL, USA). All scans were conducted by a single experienced sonographer, with at least three repeated measurements obtained and averaged. It included fetal biometry (head and abdominal circumference and femur length), which was used to estimate fetal weight using Hadlock’s equation, including an age-specific standard deviation score (SDS) calculation (Hadlock *et al.*, 1985), as well as assessment of fetal AGD. Fetal AGD was measured in the axial plane from the centre of the anus to the posterior base of the scrotum (Fischer *et al.*, 2024a).

### Child examination

Obstetrical outcomes were retrieved from the mothers’ medical records. Birth weight was converted to sex- and gestational age (GA)-specific SDS using Nordic reference data, as described by Maršál *et al.* (1996). The children were examined during minipuberty (mean (SD) age 3.1 ± 0.4 months), including weight and length measurements. AGD was measured as the ano-scrotal (AGDas) distance and ano-penile (AGDap) distance using the standardized protocol developed in The Infant Development and the Environment Study (TIDES) (Sathyanarayana *et al.*, 2015). Testicular volume was assessed by ultrasound (Hitachi Aloka Nobulus, Santax Medico) and calculated from length (a), width (b), and depth (c) (π/6*abc). Volumes were calculated for each testis, and the mean of the left and right testicular volumes was used for analyses. In addition, penile width and length (flaccid and stretched penile length) were measured using a dial caliper to the nearest millimetre (Wiha, dialMax Calliper).

### Serum samples

Blood samples were drawn from an antecubital vein during the study examination, and serum concentrations of reproductive hormones were measured (n = 247/287, 85%). The total blood volume obtained at each examination was restricted to a maximum of 1.5 ml/kg body weight in accordance with international recommendations (Howie, 2011). Samples were clotted, centrifuged, and serum was stored at −20°C until analyses.

Sixteen steroid metabolites were quantified using an in-house isotope-dilution online TurboFlow LC-MS/MS method (Frederiksen *et al.*, 2024). These comprised three progestins (progesterone (PROG), 17-hydroxypregnenolone (17-OHPreg), and 17-hydroxyprogesterone (17-OHP)); three mineralocorticoids (11-deoxycorticosterone (DOC), corticosterone (CORT), and aldosterone (ALDO)); four glucocorticoids (11-deoxycortisol (11-DOC), 21-deoxycortisol (21-DOC), cortisol, cortisone); five androgens (dehydroepiandrosterone (DHEA), dehydroepiandrosterone sulfate (DHEAS), androstenedione (Adione), testosterone (T), and dihydrotestosterone (DHT)), and the estrogen estrone sulfate (E1-S). Calibrators and 3 × 3 quality controls were included in all analytical batches. 21-DOC, DHEA, and E1-S were below the respective limits of detection (LODs) in the majority of the samples and therefore excluded from further analyses. Free T was based on the ratio between T and measured Sexual Hormone Binding Globulin (SHBG) using the Vermeulen formula (Vermeulen *et al.*, 1999). Anti-Müllerian hormone (AMH), FSH, LH, and inhibin B were measured by immunoassays. Additional methodological details are provided in the cohort profile (Fischer *et al.*, 2024b). The LODs and analytical methods of blood samples are presented in Supplementary Table S1. Limited serum volumes occasionally precluded complete hormone profiling.

### Exposure to paracetamol

Exposure windows during pregnancy were predefined based on the timing of the MPW. Prenatal paracetamol exposure was categorized as any exposure during pregnancy (n = 137) versus unexposed (n = 150). Early exposure was defined as exposure at a GA <15 weeks (n = 81). Late exposure was defined as exposure at GA ≥15 weeks (n = 118). Nineteen were exclusively exposed during early fetal life, 56 infants exclusively during late fetal life, and 62 were exposed during both early and late fetal life.

### Outcomes

Primary outcomes were predefined as main markers of testicular function and fetal androgen action: testicular volume, AGD, and circulating testosterone levels. Additional outcomes included related parameters, such as penile size, and circulating concentrations of AMH, FSH, LH, inhibin B, and steroid intermediates of testosterone and DHT. These additional parameters were included to characterize different aspects of testicular function and steroidogenesis and to facilitate interpretation of potential biological pathways, rather than to serve as independent primary outcomes.

### Statistics

For descriptive statistics, continuous variables are presented as mean (SD), and categorical variables as numbers and percentages. The distribution of testicular volume was mildly right-skewed without extreme outliers; however, for consistency with subsequent linear regression analyses, all outcomes are reported as mean (SD).

Correlations between testicular volume and circulating levels of reproductive hormones were assessed by Pearson correlation coefficients.

Differences in maternal and infant characteristics between exposure groups were assessed using Student’s *t*-test. For categorical variables, Pearson’s chi-square test with Yates’ continuity correction was applied when appropriate; Fisher’s exact test was used when expected cell counts were below 5.

Circulating steroid hormone concentrations were transformed into age- and sex-specific SD scores using the published L (skewness), M (median), and S (coefficient of variation) parameters derived from the Copenhagen Minipuberty Study, a longitudinal cohort of healthy Danish infants (ClinicalTrials.gov NCT01411527) (Busch *et al.*, 2021). The LMS parameters were estimated using generalized additive models for location, scale, and shape (GAMLSS), implemented in the GAMLSS R package (Stasinopoulos and Rigby, 2007). SD scores for the present study were calculated according to the formula SD score = ((*X*/*M*)  ^  *L* − 1)/(*L* × *S*), where *X* denotes the observed concentration and *L* ≠ 0.

#### Exposure at any time during fetal life

Associations between prenatal paracetamol exposure and outcomes were assessed using linear regression models comparing any exposure during pregnancy with unexposed controls. Regression coefficients (β) and 95% CI were estimated for each outcome in both unadjusted and adjusted models. Model assumptions were evaluated using residual and Q–Q plots.

Adjusted models included maternal pre-pregnancy BMI, maternal smoking, and alcohol use up to gestational week 10, maternal use of other mild analgesics during pregnancy (acetylsalicylic acid (ASA) and non-steroidal anti-inflammatory drugs (NSAIDs)), and the child’s use of paracetamol from birth until the clinical examination. These covariates were selected *a priori* based on existing literature, as factors known or suspected to influence fetal growth, gonadal development, or steroid hormone synthesis. Maternal use of ASA and NSAIDs was included to account for potential confounding by other commonly used analgesics with cyclooxygenase-inhibiting properties that may also influence fetal reproductive development.

To account for physiological scaling, fetal or postnatal body size was additionally included as a covariate in all AGD regression analyses.

#### Exposure according to timing during fetal life

To assess effects of exposure according to timing during fetal life (early fetal life, late fetal life, and combined early- and late fetal life), we used multiple linear regression:


$$Y=\alpha +\beta_{\mathrm{Early}}+\beta_{\mathrm{Late}}+\text{adjusting}\;\text{variables}+\epsilon$$

Early and late exposures were included simultaneously in the model, allowing estimation of their independent effects. Specifically, the effect of early exposure was estimated while holding late exposure constant, and vice versa. Thus, the estimated effect of early exposure reflects a comparison between individuals with and without early exposure who have the same late exposure status, and similarly for late exposure.

In this additive model, the combined effect of exposure in both periods corresponds to the sum of the estimated early and late exposure effects. The assumption of additivity was evaluated by including an interaction, an early-by-late interaction term in the model.

All models were adjusted as described above. Sample sizes varied slightly across outcomes due to missing data.

#### Sensitivity analyses

Maternal fever as an indication for use of paracetamol as well as maternal PCOS were not adequately frequent to be included as covariates in the multiple regression analyses (n = 29 and 11, respectively). To test potential confounding by indication, separate sensitivity analyses were performed after excluding boys of mothers reporting fever as the indication for paracetamol use, and boys of mothers reporting PCOS, respectively.

Maternal PCOS is associated with an altered hormonal milieu during pregnancy that could influence fetal reproductive development independently of paracetamol exposure.

#### Analyses according to cumulative paracetamol dose

To further explore potential dose–response relationships, two additional dose-group analyses were performed. First, cumulative paracetamol dose across pregnancy was calculated for all exposed women, irrespective of exposure timing (any exposure). Boys prenatally unexposed to paracetamol served as the reference group, while exposed boys were divided into tertiles according to the distribution of cumulative maternal dose. Associations were estimated for testicular volume, fetal AGD, AGDas, and testosterone levels.

Second, an analogous analysis was restricted to pregnancies with combined early and late paracetamol exposure. Within this subgroup, exposed boys were likewise divided into tertiles according to cumulative maternal dose, with unexposed boys serving as the reference group. Both analyses were adjusted for the same covariates as the primary analyses.

All statistical tests were two-sided, with *P*-values < 0.05 considered statistically significant. *P*-values between 0.05 and 0.10 were interpreted as indicative of trends. Analyses were performed using RStudio (version 2024.04.2; RStudio Team, Boston, MA, USA) (R Core Team, 2024).

## Results

In total, 137/287 (47.7%) of pregnant women carrying male fetuses reported using paracetamol at least once during pregnancy. Of the 448 reported paracetamol-use episodes, the most common indications were headache (68.5%) and musculoskeletal pain (15.2%), followed by general malaise (7.6%), fever (7.1%), and other (1.6%).

Among exposed pregnancies, the median (interquartile range (IQR)) cumulative prenatal paracetamol dose was 4.0 g (2.0–10.0). Mothers of boys exposed during both early and late fetal life had a higher median cumulative dose of 8.5 g (4.0–16.6). Exposure to ASA and other NSAIDs was reported in 22/287 (7.7%) and 17/287 (5.9%) boys, respectively. During the postnatal period from birth until follow-up, 35/287 (12.3%) boys received paracetamol.

Maternal age and alcohol use during the first trimester were slightly higher among boys exposed to paracetamol at any time during pregnancy compared with unexposed boys. Otherwise, no differences in maternal or infant baseline characteristics were observed between the exposure groups (Table 1). Outcome measures in boys are summarized according to prenatal paracetamol exposure at any time during pregnancy and combined early and late exposure (Supplementary Table S2).

**Table 1.** Maternal and infant characteristics by prenatal paracetamol exposure.

	Total		Unexposed		Exposed any time during pregnancy		Combined early and late exposure	
	n		n		n		n	
**Maternal characteristics**								
Age at delivery (years)	287	33.0 (4.6)	150	32.4 (3.9)	137	33.7 (5.1)*	62	33.4 (4.9)
BMI before pregnancy (kg/m^2^)	282	22.5 (2.8)	145	22.3 (2.7)	137	22.8 (2.9)	62	22.9 (3.1)
Nulliparous	287	215 (74.9%)	150	117 (78.0%)	137	98 (71.5%)	62	40 (64.5%)
Nicotine use in first trimester of pregnancy (yes)	287	13 (4.5%)	150	7 (4.7%)	137	6 (4.4%)	62	2 (3.2%)
Alcohol use in first trimester of pregnancy (yes)	285	89 (31.2%)	148	42 (28.4%)	137	47 (34.3%)*	62	16 (25.8%)
Polycystic ovarian syndrome	279	11 (3.9%)	148	6 (4.1%)	131	5 (3.8%)	62	3 (4.8%)
Cesarean section (emergency)	287	32 (11.1%)	150	17 (11.3%)	137	15 (10.9%)	62	6 (9.7%)
IUI	285	24 (8.4%)	148	14 (9.5%)	137	10 (7.3%)	62	5 (8.1%)
IVF	285	12 (4.2%)	148	5 (3.4%)	137	7 (5.1%)	62	2 (3.2%)
ICSI	285	8 (2.8%)	148	3 (2.0%)	137	5 (3.6%)	62	1 (1.6%)
**Infant characteristics**								
Estimated Fetal Weight (g)	287	1884 (328)	150	1871 (305)	137	1897 (352)	62	1933 (398)
Estimated Fetal Weight deviation (%)	287	−0.1 (7.4)	150	−0.6 (7.1)	137	0.4 (7.8)	62	1.2 (7.5)
Gestational age at delivery (days)	287	282 (8)	150	282 (8)	137	282 (8)	62	281 (6.7)
Birth weight (g)	284	3666 (427)	148	3661 (430)	136	3672 (426)	61	3643 (390)
Birth weight (SDS)	284	0.3 (0.9)	148	0.3 (0.9)	136	0.3 (0.9)	61	0.2 (0.9)
Weight at child examination (kg)	287	6.5 (0.8)	150	6.5 (0.8)	137	6.6 (0.7)	62	6.6 (0.7)
Weight at child examination (SDS)	287	−0.01 (0.7)	150	−0.05 (0.6)	137	0.03 (0.7)	62	0.1 (0.7)

SDS, standard deviation score. Continuous variables: mean (SD). Binary variables: n (%). *P* values were calculated using the Student’s test or Pearson chi-square test, as appropriate. Continuous variables: mean (SD); n shown. Binary variables: n (%). *P*-values compare unexposed boys with boys exposed at any time during pregnancy, and with boys exposed during both early and late fetal life.

* indicates a *P*-value < 0.05.

In the cohort, only two cases of hypospadias (first degree, coronal) and three cases of cryptorchidism (one bilateral, inguinal, and two unilateral, inguino-scrotal) were identified – not sufficient for statistical evaluations.

### Correlations between testicular volume and circulating levels of reproductive hormones

Testicular volume at infancy correlated negatively with FSH (*r* = −0.14, *P* = 0.030) and LH (*r* = −0.20, *P* = 0.002), and positively with inhibin B (*r* = 0.26, *P* < 0.001), and AMH (*r* = 0.18, *P* = 0.005), but not with circulating levels of testosterone (*r* = −0.08, *P* = 0.243).

### Associations between prenatal paracetamol exposure and outcomes in infancy

In univariate analyses as well as after adjustment for relevant covariates, prenatal exposure to paracetamol at any time during pregnancy was associated with smaller testicular volume (β) −25.4 mm³, 95% CI −50.3 to −0.4, *P* = 0.046. No other primary outcomes were associated with prenatal exposure. In secondary analyses, prenatal paracetamol exposure was associated with lower levels of steroid intermediates: PROG, 17-OHPreg, and 17-OHP, 11-DOC, and Adione compared with unexposed boys (Table 2).

**Table 2.** Univariate and multiple linear regression analyses of reproductive and steroid hormone outcomes according to prenatal paracetamol exposure at any time during pregnancy versus no exposure.

	Exposed any time during pregnancy			
	Unadjusted		Adjusted for covariates	
**Primary outcomes**	n		n	
Testicular volume (mm³)	130	−26.2 (−50.1; −2.3)*	127	−25.4 (−50.3; −0.4)*
Fetal AGD (mm)	134	−0.43 (−1.13; 0.28)	131	−0.37 (−1.11; 0.38)
AGDas (mm)	137	0.08 (−1.17; 1.33)	134	0.02 (−1.28; 1.33)
T (SDS)	118	−0.02 (−0.24; 0.21)	115	0.03 (−0.20; 0.26)
**Secondary outcomes**				
AGDap (mm)	137	−0.56 (−2.20; 1.07)	134	−0.44 (−2.09; 1.21)
Penile width (mm)	137	−0.16 (−0.55; 0.23)	134	−0.20 (−0.60; 0.20)
Penile length (mm)	137	0.34 (−0.68; 1.35)	134	0.40 (−0.66; 1.46)
LH (SDS)	115	−0.01 (−0.20; 0.17)	112	−0.01 (−0.20; 0.19)
FSH (SDS)	114	−0.12 (−0.39; 0.14)	111	−0.10 (−0.38; 0.18)
LH/FSH ratio	114	0.12 (−0.07; 0.32)	111	0.09 (−0.10; 0.29)
LH/T ratio	115	−0.02 (−0.09; 0.06)	112	−0.03 (−0.11; 0.05)
AMH (SDS)	116	0.18 (−0.07; 0.42)	113	0.10 (−0.15; 0.35)
Inhibin B (SDS)	113	0.00 (−0.24; 0.24)	110	−0.02 (−0.28; 0.23)
SHBG (SDS)	111	−0.12 (−0.37; 0.14)	108	−0.08 (−0.34; 0.19)
PROG (SDS)	118	−0.38 (−0.65; −0.12)*	115	−0.36 (−0.65; −0.08)*
17-OHPreg (SDS)	118	−0.32 (−0.60; −0.04)*	115	−0.32 (−0.62; −0.02)*
17-OHP (SDS)	118	−0.35 (−0.58; −0.13)*	115	−0.34 (−0.58; −0.11)*
Free T (pmol/l)	111	−0.37 (−3.00; 2.27)	108	0.00 (−2.78; 2.78)
DHEAS (SDS)	118	−0.22 (−0.57; 0.13)	115	−0.29 (−0.66; 0.08)
Adione (SDS)	118	−0.27 (−0.55; 0.01)	115	−0.30 (−0.59; −0.01)*
DHT (SDS)	118	0.04 (−0.14; 0.23)	115	0.08 (−0.11; 0.27)
DOC (SDS)	118	−0.14 (−0.39; 0.12)	115	−0.15 (−0.41; 0.10)
CORT (SDS)	118	−0.11 (−0.36; 0.13)	115	−0.15 (−0.41; 0.11)
Aldo (SDS)	118	−0.07 (−0.27; 0.13)	115	−0.05 (−0.26; 0.17)
11-DOC (SDS)	118	−0.24 (−0.51; 0.04)	115	−0.29 (−0.58; −0.01)*
Cortisol (SDS)	118	−0.10 (−0.32; 0.12)	115	−0.15 (−0.38; 0.08)
Cortisone (SDS)	118	−0.07 (−0.31; 0.16)	115	−0.10 (−0.35; 0.15)

Data are presented as β coefficients with 95% confidence intervals from unadjusted and adjusted linear regression models. Exposure was analysed as any prenatal paracetamol exposure with unexposed as the reference group. DOC, 11-deoxycorticosterone; 11-DOC, 11-deoxycortisol, 17-OHPreg, 17-hydroxypregnenolone; 17-OHP, 17-hydroxyprogesterone; ALDO, aldosterone; Adione, androstenedione; AMH, anti-Müllerian hormone; AGD, ano-genital distance; AGDas, ano-scrotal anogenital distance; AGDap, ano-penile anogenital distance; CORT, corticosterone; DHEAS, dehydroepiandrosterone sulfate; DHT, dihydrotestosterone; PROG, progesterone; SHBG, sex hormone binding globulin; T, testosterone. Adjusted models include maternal pre-pregnancy BMI, maternal nicotine and alcohol use in the first trimester of pregnancy, maternal acetylsalicylic acid (ASA) and non-steroidal anti-inflammatory drugs (NSAIDs) use during pregnancy, and child paracetamol use. Analyses of fetal AGD were additionally body parameter adjusted for estimated fetal weight, and analyses of AGDas and AGDap were additionally body parameter adjusted for body weight at examination.

* indicates a *P*-value < 0.05.

To further explore potential dose–response relationships, cumulative prenatal paracetamol dose across pregnancy was categorized into tertiles among exposed pregnancies and compared with unexposed boys. The tertiles comprised 46, 46, and 45 boys, with median (IQR) cumulative doses of 1.0 g (1.0–1.9), 4.0 g (3.1–6.0), and 13.8 g (10.0–20.0), respectively. This analysis did not demonstrate a consistent monotonic dose–response pattern for the predefined primary outcomes. Negative effect estimates for testicular volume were observed in the first and third exposure tertiles, whereas the second tertile demonstrated estimates close to the null. Likewise, no consistent dose-related patterns were observed for fetal AGD, AGDas, or testosterone (Supplementary Fig. S2).

Furthermore, boys exposed during both early and late fetal life exhibited the largest negative estimates for testicular volume; (β) −34.1 mm³, 95% CI −65.5 to −2.7, *P* = 0.033, and circulating levels of PROG, 17-OHPreg, and 17-OHP, as well as a trend towards shorter fetal AGD; −0.85 mm, −1.79 to 0.10, *P* = 0.079 compared with unexposed boys (Table 3, Fig. 1). No statistically significant interaction between early and late exposure was observed across outcomes (Supplementary Table S3).

**Figure 1.**
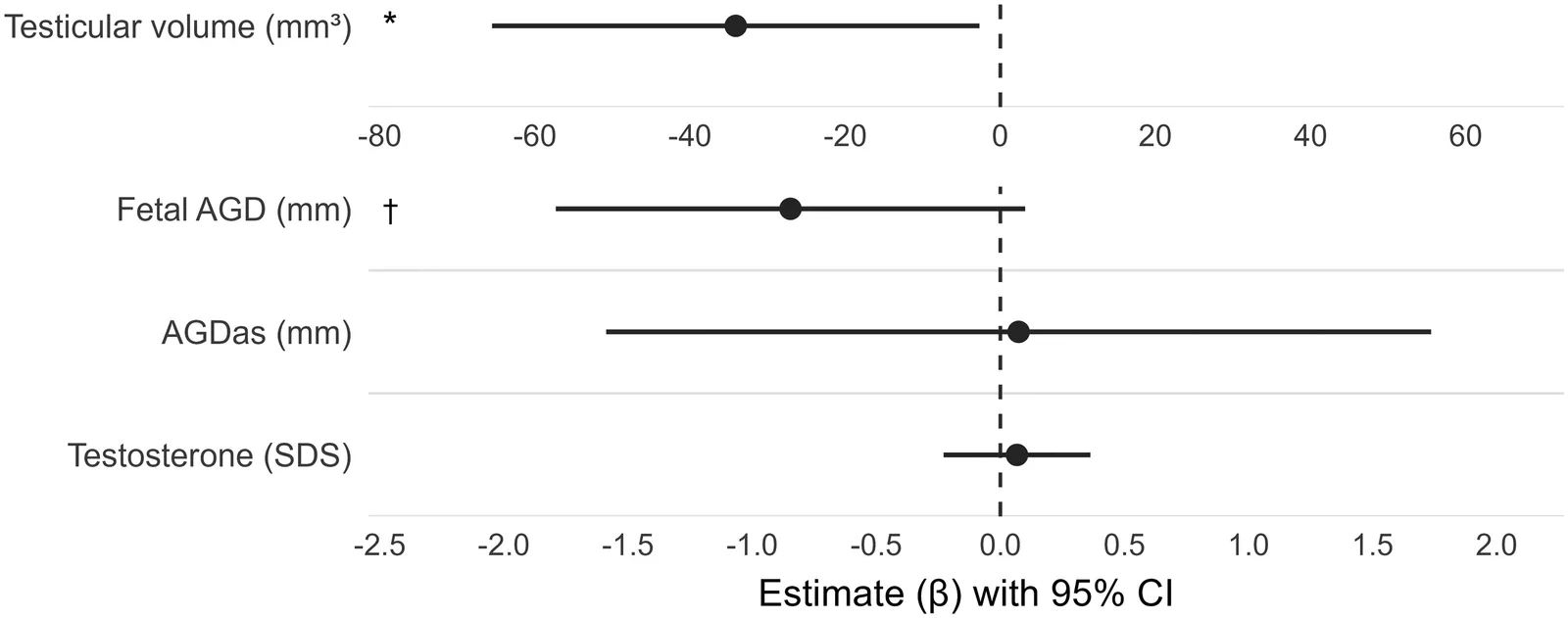
**Associations between combined early and late prenatal paracetamol exposure and primary reproductive outcomes.** Adjusted estimates (β) with 95% confidence intervals for testicular volume, fetal anogenital distance (AGD), postnatal anogenital distance from the anus to the scrotum (AGDas), and testosterone following combined early and late prenatal paracetamol exposure. Testosterone was expressed as age-adjusted standard deviation scores (SDS). The upper *x*-axis (−80 to 60) applies to testicular volume (mm³), whereas the lower *x*-axis (−2.5 to 2.0) applies to fetal AGD (mm), AGDas (mm), and testosterone (SDS). Estimates for combined exposure were derived from an additive regression model including separate terms for early and late exposure, with the combined effect calculated as the sum of the early and late regression coefficients. Models were adjusted for maternal pre-pregnancy BMI, maternal smoking and alcohol use in early pregnancy, maternal use of acetylsalicylic acid and non-steroidal anti-inflammatory drugs during pregnancy, and postnatal paracetamol use by the child. The fetal AGD model was additionally adjusted for estimated fetal weight, and the postnatal AGDas model for body weight at examination. *indicates a *P*-value < 0.05, ^†^indicates 0.05 < *P* ≤ 0.10.

**Table 3.** Multiple linear regression analyses of reproductive and steroid hormone outcomes according to prenatal paracetamol exposure.

	Early exposure				Late exposure				Combined early and late exposure			
	Adjusted for late exposure		Adjusted for additional covariates		Adjusted for early exposure		Adjusted for additional covariates		Sum of early and late exposure§		Sum of early and late exposure adjusted for additional covariates	
**Primary outcomes**	n		n		n		n		n		n	
Testicular volume (mm³)	79	−23.2 (−53.0; 6.7)	76	−24.3 (−55.0; 6.5)	111	−12.2 (−39.7; 15.3)	109	−9.8 (−37.7; 18.0)	60	−35.4 (−65.2; −5.6)*	58	−34.1 (−65.5; −2.7)*
Fetal AGD (mm)	79	−0.46 (−1.49; 0.58)	76	−0.55 (−1.46; 0.37)	115	−0.07 (−1.01; 0.88)	113	−0.30 (−1.12; 0.52)	60	−0.52 (−1.57; 0.52)	58	−0.85 (−1.79; 0.10)
AGDas (mm)	81	−0.28 (−1.92; 1.36)	78	−0.28 (−1.90; 1.33)	118	0.62 (−0.87; 2.12)	116	0.36 (−1.09; 1.81)	62	0.34 (−1.30; 1.99)	60	0.07 (−1.59; 1.74)
T (SDS)	69	−0.01 (−0.28; 0.26)	66	0.05 (−0.23; 0.33)	101	0.01 (−0.24; 0.25)	99	0.02 (−0.24; 0.27)	52	−0.01 (−0.28; 0.27)	50	0.07 (−0.23; 0.36)
**Secondary outcomes**												
AGDap (mm)	81	−0.72 (−2.95; 1.52)	78	−0.61 (−2.64; 1.43)	118	0.63 (−1.41; 2.68)	116	0.37 (−1.47; 2.20)	62	−0.08 (−2.33; 2.17)	60	−0.24 (−2.34; 1.86)
Penile width (mm)	81	−0.19 (−0.67; 0.29)	78	−0.20 (−0.69; 0.30)	118	−0.04 (−0.48; 0.40)	116	−0.09 (−0.53; 0.36)	62	−0.23 (−0.72; 0.25)	60	−0.29 (−0.80; 0.22)
Penile length (mm)	81	0.48 (−0.78; 1.74)	78	0.59 (−0.71; 1.90)	118	0.13 (−1.02; 1.28)	116	0.20 (−0.97; 1.38)	62	0.61 (−0.65; 1.88)	60	0.80 (−0.55; 2.14)
LH (SDS)	68	0.09 (−0.14; 0.32)	65	0.13 (−0.11; 0.37)	98	−0.07 (−0.28; 0.13)	96	−0.08 (−0.30; 0.13)	51	0.01 (−0.22; 0.24)	49	0.05 (−0.21; 0.30)
FSH (SDS)	68	−0.11 (−0.44; 0.22)	65	−0.01 (−0.35; 0.33)	97	−0.04 (−0.34; 0.27)	95	−0.12 (−0.42; 0.19)	51	−0.14 (−0.48; 0.19)	49	−0.13 (−0.48; 0.23)
LH/FSH ratio	68	0.20 (−0.03; 0.44)	65	0.14 (−0.10; 0.38)	97	−0.02 (−0.23; 0.20)	95	0.05 (−0.17; 0.27)	51	0.19 (−0.05; 0.43)	49	0.19 (−0.06; 0.44)
LH/T ratio	68	0.04 (−0.05; 0.13)	65	0.04 (−0.06; 0.13)	98	−0.04 (−0.13; 0.04)	96	−0.05 (−0.14; 0.03)	51	0.00 (−0.09; 0.09)	49	−0.02 (−0.12; 0.09)
AMH (SDS)	69	0.08 (−0.22; 0.38)	66	−0.04 (−0.35; 0.27)	99	0.13 (−0.15; 0.40)	97	0.17 (−0.11; 0.45)	52	0.21 (−0.10; 0.51)	50	0.13 (−0.19; 0.45)
Inhibin B (SDS)	69	−0.27 (−0.57; 0.02)	66	−0.32 (−0.63; −0.01)*	96	0.20 (−0.07; 0.47)	94	0.23 (−0.05; 0.51)	52	−0.07 (−0.37; 0.23)	50	−0.09 (−0.41; 0.22)
SHBG (SDS)	67	0.13 (−0.18; 0.45)	64	0.22 (−0.11; 0.55)	96	−0.20 (−0.49; 0.10)	94	−0.22 (−0.51; 0.08)	52	−0.06 (−0.38; 0.25)	50	0.00 (−0.33; 0.33)
PROG (SDS)	69	−0.08 (−0.41; 0.24)	66	−0.06 (−0.41; 0.28)	101	−0.44 (−0.74; −0.14)*	99	−0.42 (−0.73; −0.11)*	52	−0.52 (−0.86; −0.19)*	50	−0.48 (−0.85; −0.12)*
17-OHPreg (SDS)	69	−0.16 (−0.51; 0.18)	66	−0.17 (−0.54; 0.19)	101	−0.26 (−0.58; 0.05)	99	−0.26 (−0.59; 0.07)	52	−0.43 (−0.78; −0.07)*	50	−0.43 (−0.81; −0.05)*
17-OHP (SDS)	69	−0.13 (−0.41; 0.15)	66	−0.12 (−0.41; 0.17)	101	−0.30 (−0.56; −0.05)*	99	−0.28 (−0.55; −0.02)*	52	−0.43 (−0.72; −0.15)*	50	−0.40 (−0.71; −0.10)*
Free T (pmol/l)	67	−1.85 (−5.15; 1.45)	64	−1.42 (−4.88; 2.03)	96	0.79 (−2.24; 3.82)	94	0.94 (−2.17; 4.05)	52	−1.06 (−4.32; 2.20)	50	−0.48 (−3.97; 3.02)
DHEAS (SDS)	69	−0.21 (−0.64; 0.23)	66	−0.28 (−0.73; 0.17)	101	−0.03 (−0.43; 0.37)	99	−0.11 (−0.52; 0.29)	52	−0.24 (−0.68; 0.21)	50	−0.39 (−0.86; 0.08)
Adione (SDS)	69	−0.13 (−0.47; 0.21)	66	−0.16 (−0.52; 0.20)	101	−0.17 (−0.48; 0.14)	99	−0.19 (−0.51; 0.13)	52	−0.30 (−0.65; 0.04)	50	−0.35 (−0.73; 0.02)
DHT (SDS)	69	0.05 (−0.18; 0.28)	66	0.11 (−0.12; 0.34)	101	0.01 (−0.20; 0.22)	99	0.01 (−0.20; 0.22)	52	0.06 (−0.17; 0.29)	50	0.12 (−0.12; 0.37)
DOC (SDS)	69	0.06 (−0.25; 0.37)	66	0.08 (−0.23; 0.40)	101	−0.18 (−0.47; 0.11)	99	−0.18 (−0.47; 0.10)	52	−0.12 (−0.44; 0.20)	50	−0.10 (−0.43; 0.23)
CORT (SDS)	69	0.05 (−0.26; 0.35)	66	0.05 (−0.27; 0.36)	101	−0.14 (−0.42; 0.14)	99	−0.15 (−0.43; 0.13)	52	−0.09 (−0.40; 0.22)	50	−0.10 (−0.43; 0.23)
Aldo (SDS)	69	−0.02 (−0.27; 0.23)	66	0.06 (−0.20; 0.32)	101	−0.04 (−0.27; 0.19)	99	−0.05 (−0.29; 0.18)	52	−0.06 (−0.31; 0.20)	50	0.00 (−0.27; 0.28)
11-DOC (SDS)	69	−0.09 (−0.43; 0.25)	66	−0.13 (−0.48; 0.22)	101	−0.19 (−0.50; 0.12)	99	−0.20 (−0.51; 0.12)	52	−0.28 (−0.62; 0.07)	50	−0.32 (−0.69; 0.04)
Cortisol (SDS)	69	−0.01 (−0.29; 0.26)	66	−0.05 (−0.34; 0.24)	101	−0.10 (−0.35; 0.15)	99	−0.13 (−0.38; 0.13)	52	−0.12 (−0.40; 0.17)	50	−0.18 (−0.48; 0.12)
Cortisone (SDS)	69	−0.18 (−0.46; 0.11)	66	−0.22 (−0.52; 0.09)	101	0.08 (−0.18; 0.34)	99	0.08 (−0.19; 0.35)	52	−0.10 (−0.39; 0.20)	50	−0.14 (−0.45; 0.18)

Data are presented as β coefficients with 95% confidence intervals from unadjusted and adjusted linear regression models. Exposure was analysed using a multi-category model including early pregnancy exposure and late pregnancy exposure with unexposed as the reference group. Combined early + late exposure represents the sum of the estimates for early and late exposure, where §early exposure is adjusted for late, and late exposure is adjusted for early. DOC, 11-deoxycorticosterone; 11-DOC, 11-deoxycortisol; 17-OHPreg, 17-hydroxypregnenolone; 17-OHP, 17-hydroxyprogesterone; ALDO, aldosterone; Adione, androstenedione; AMH, anti-Müllerian hormone; AGD, ano-genital distance; AGDas, ano-scrotal anogenital distance; AGDap, ano-penile anogenital distance; CORT, corticosterone; DHEAS, dehydroepiandrosterone sulfate; DHT, dihydrotestosterone; PROG, progesterone; SHBG, sex hormone-binding globulin; SDS, standard deviation score; T, testosterone. Adjusted models include maternal pre-pregnancy BMI, maternal nicotine and alcohol use in the first trimester of pregnancy, maternal ASA and NSAID use during pregnancy, and child paracetamol use. Analyses of fetal AGD were additionally body parameter adjusted for estimated fetal weight, and analyses of AGDas and AGDap were additionally body parameter adjusted for body weight at examination.

* indicates a *P*-value < 0.05.

Among pregnancies with combined early and late exposure, cumulative maternal paracetamol dose was likewise categorized into tertiles. The tertiles comprised 21, 21, and 20 boys, with median (IQR) cumulative doses of 3.5 g (3.0–4.0), 9.0 g (6.5–10.5), and 19.5 g (17.0–25.3), respectively. Testicular volume was affected from exposure tertile 2, but further dose–response effect in the highest exposed group was not evident. Likewise, no consistent dose-related patterns were observed for fetal AGD, AGDas, or testosterone (Fig. 2).

**Figure 2.**
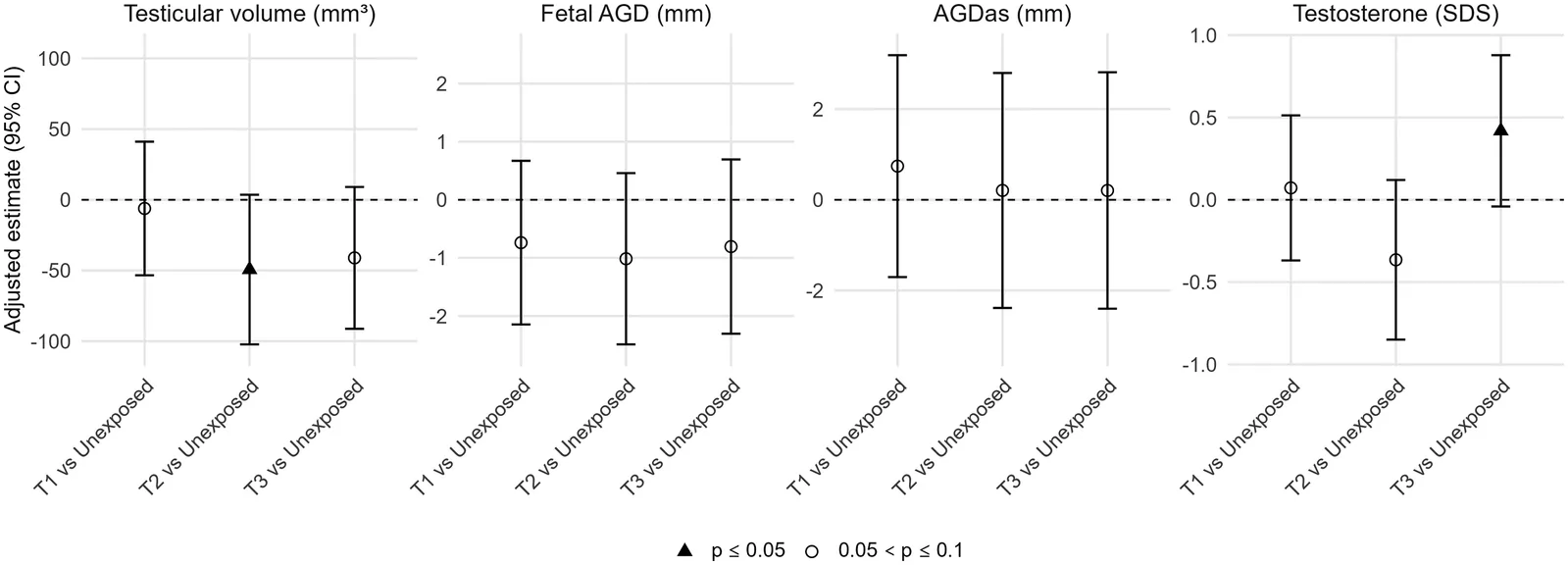
**Associations between cumulative prenatal paracetamol dose and primary reproductive outcomes among pregnancies with combined early and late exposure.** Adjusted differences in testicular volume, fetal anogenital distance (AGD), postnatal anogenital distance from the anus to the scrotum (AGDas), and testosterone levels, expressed as age-adjusted standard deviation scores (SDS), according to cumulative maternal paracetamol dose among pregnancies with paracetamol exposure during both early and late fetal life. Prenatally unexposed boys served as the reference group, and boys with combined early and late exposure were divided into tertiles (T1 − T3) according to the distribution of cumulative dose within this exposure group. Estimates are presented with 95% confidence intervals. Models were adjusted for maternal smoking and alcohol use in early pregnancy, maternal pre-pregnancy BMI, maternal use of acetylsalicylic acid and non-steroidal anti-inflammatory drugs during pregnancy, and postnatal paracetamol use by the child. The fetal AGD model was additionally adjusted for estimated fetal weight, and the postnatal AGDas model for body weight at examination. Filled triangles indicate associations of borderline statistical significance (0.05 < *P* ≤ 0.10) and open circles indicate non-significant associations (*P* > 0.10).

In analyses separating the exposure windows, neither early fetal exposure (GW <15 weeks, n = 81) nor late fetal exposure (GW ≥ 15, n = 118) was associated with primary outcomes. Early exposure was associated with lower levels of inhibin B; −0.32 SDS, −0.63 to −0.01, *P* = 0.040, whereas late fetal exposure was associated with lower levels of PROG and 17-OHP (Table 3).

Sensitivity analyses, excluding mothers reporting fever as the indication for using paracetamol (n = 29) and excluding mothers with PCOS (n = 11), did not affect the direction of the observed associations (Supplementary Table S4).

## Discussion

In this prospective cohort study, prenatal paracetamol exposure was associated with smaller testicular volume during minipuberty, whereas no consistent associations were observed for the remaining predefined primary outcomes. The largest negative estimate for testicular volume was observed among boys exposed during both early and late fetal life. In addition, prenatal paracetamol exposure was associated with lower circulating levels of several steroid intermediates, including PROG, 17-OHPreg, 17-OHP, 11-DOC, and Adione.

Early prenatal exposure alone was associated with an estimated 24 mm³ lower testicular volume compared with no exposure, although this estimate was not statistically significant. Additional exposure during late pregnancy was associated with a further estimated difference of approximately −10 mm³, yielding an overall estimated difference of −34 mm³ among boys exposed during both early and late fetal life. Overall, the estimated difference of −34 mm³ (−0.034 ml) corresponds to an average reduction of 8% compared with the mean testicular volume among unexposed boys in our cohort. In adults, testicular volume is an established marker of testicular function and correlates positively with total sperm count, semen quality, and circulating levels of reproductive hormones (Takihara *et al.*, 1987). Whether testicular volume during infancy predicts adult reproductive function remains uncertain. However, a contemporary longitudinal birth cohort demonstrated a positive, although non-significant, association between testicular volume measured during minipuberty and total sperm count in adulthood, suggesting that infant testicular size may reflect later fertility potential (Scheutz Henriksen *et al.*, 2022).

In infancy, testicular volume primarily reflects Sertoli cell number (Cortes *et al.*, 1987; Rey *et al.*, 1993; Nistal *et al.*, 2015). In support, testicular volume in infant boys was associated with FSH and Sertoli cell products (inhibin B and AMH) but not with circulating levels of testosterone. The lower levels of inhibin B following early prenatal exposure to paracetamol suggest that exposure could affect Sertoli cell function. We speculate that Sertoli cell proliferation and differentiation may be disrupted by paracetamol directly through inhibition of cell-cycle progression and/or through disruption of prostaglandin-dependent processes (Rossitto *et al.*, 2019; Corpuz-Hilsabeck *et al.*, 2023; Nielsen *et al.*, 2025).

Based on fetal differentiation of somatic cells in the developing testes, we suggest a hypothesis linking our present findings with existing evidence of the anti-androgenic properties of paracetamol. Experimental studies in rodents and *ex vivo* human testicular tissue indicate that fetal exposure to paracetamol impairs Leydig cell steroidogenesis by inhibiting key cytochrome P450 enzymes, including CYP11A1 and CYP17A1 (van den Driesche *et al.*, 2015). These enzymes are essential for the conversion of cholesterol into pregnenolone and subsequent androgen steroid intermediates, potentially leading to reduced testosterone synthesis (van den Driesche *et al.*, 2015). While we did not observe associations between prenatal paracetamol exposure and infant testosterone concentrations, the upstream steroid intermediates (PROG, 17-OHPreg, 17-OHP, and Adione), were lower in exposed boys. We speculate that this may reflect reduced availability of steroid intermediates during fetal life, when androgen signaling is essential for masculinization. Reduced substrate availability therefore provides a plausible mechanistic link to the observed trend towards shorter fetal AGD in the present study, and the shorter postnatal AGD reported in other cohort and experimental studies (Kristensen *et al.*, 2011; Holm *et al.*, 2015; Fisher *et al.*, 2016; Lind *et al.*, 2017; van den Driesche *et al.*, 2017; Andreasen *et al.*, 2025). Although epidemiological evidence linking prenatal paracetamol exposure with male reproductive outcomes remains heterogeneous (Eletri *et al.*, 2026), the anti-androgenic effect of paracetamol is supported by consistent associations to cryptorchidism across epidemiological studies (Berkowitz and Lapinski, 1996; Rebordosa *et al.*, 2008; Jensen *et al.*, 2010; Kristensen *et al.*, 2011; Wagner-Mahler *et al.*, 2011; Snijder *et al.*, 2012; Fisher *et al.*, 2016; Zafeiri *et al.*, 2022). Fetal Leydig cell function is essential for testicular descent, relying not only on androgen production and action but also on INSL3 signaling, which is required for proper development of the gubernaculum. A potential association with cryptorchidism may therefore indicate more broadly impaired Leydig cell function rather than an isolated impairment of cytochrome P450-dependent androgen synthesis. Sertoli cells play a central role in orchestrating fetal testis development (Sanou *et al.*, 2026), and it is biologically plausible that subtle, generalized Leydig cell dysfunction could arise, at least in part, secondary to altered Sertoli cell signaling. This framework provides a potential link between the reduced testicular volume and lower circulating levels of inhibin B observed in the present study with established clinical markers of impaired fetal Leydig cell function, including reduced AGD and increased risk of cryptorchidism.

Intensified effect size of prolonged prenatal exposure is in line with observations from previous studies (Jensen *et al.*, 2010; Kristensen *et al.*, 2012; van den Driesche *et al.*, 2015). We found no evidence of interaction between early and late exposure, suggesting that the association observed for combined exposure can be explained by the sum of the independent contributions of early and late exposure rather than a synergistic effect, and therefore, an additive model appears appropriate to describe the impact of exposure in this cohort. These findings could suggest that effects were related to the accumulated paracetamol dose; however, specific analyses did not reveal a consistent dose-dependent pattern of the primary outcomes in the present cohort. Our findings are compatible with continued susceptibility of steroidogenesis and testicular function beyond the classical MPW.

Causality cannot be established from this observational study. However, the observed associations are consistent with findings from experimental animal models and *ex vivo* human studies demonstrating adverse effects of prenatal paracetamol exposure on fetal testicular development and function, including at exposure levels comparable to recommended human doses (Kristensen *et al.*, 2012; Dean *et al.*, 2016). Residual confounding by indication cannot be excluded, although sensitivity analyses excluding women reporting fever as an indication for paracetamol use yielded similar results.

Analyses were pre-specified and hypothesis-driven, and no correction for multiple testing was applied, as the variables reflect interconnected biological processes, and strict adjustment could increase the risk of false negative findings. The cohort consists predominantly of term-born Caucasian infants, which may limit generalizability. In addition, long-term reproductive outcomes such as semen quality or testicular cancer require larger cohorts and extended follow-up, and the study was not powered to assess rare outcomes such as cryptorchidism or hypospadias. Moreover, as hormone levels were assessed based on a single blood sample during minipuberty, temporal dynamics could not be evaluated.

Current guidelines from the European Medicines Agency recommend using the lowest effective dose of paracetamol for the shortest possible duration (European Medicines Agency (EMA), 2025) and reported use in the present study was consistent with these recommendations. Although our findings add to the growing body of evidence suggesting that prenatal paracetamol exposure may influence early reproductive development, they should be interpreted in the context of the study’s observational design, which precludes causal inference, and the uncertain long-term clinical significance of the observed clinical findings at infancy. However, our study contributes to the overall evidence base regarding the safety of paracetamol use during pregnancy, complementing findings from both experimental and epidemiological research. It will be for the relevant regulatory authorities to re-evaluate if the present total body of evidence gives rise to restricted recommendations of paracetamol use during pregnancy. However, in accordance with the precautionary principle, we believe that the present findings warrant certain reflections: The most frequent indications for use of paracetamol were headache and musculoskeletal pain, which may be self-limiting or responsive to non-pharmacological approaches. The indication for maternal use of paracetamol should therefore be considered carefully. Importantly, paracetamol remains indicated in specific situations, such as high maternal fever, which is suspected to adversely affect fetal development (Dreier *et al.*, 2014; Antoun *et al.*, 2021).

## Conclusion

Prenatal paracetamol exposure was associated with smaller testicular volume and lower circulating levels of steroid intermediates in infant boys. The largest effect estimates for testicular volume were observed among boys exposed during both early and late fetal life, whereas categorized cumulative dose analyses provided no evidence of a consistent dose–response relationship.

## Supplementary Material

hoag089_Supplementary_Data

## Data Availability

The datasets generated and/or analysed during the current study are available from the corresponding author on reasonable request. The codes used for statistical analyses and figure generation are available from the corresponding author upon reasonable request.
