# Supplementary material for: Prenatal exposure to paracetamol is associated with smaller testicular volume: a COPANA cohort study of 287 healthy infant boys
Source: Hum Reprod Open. 2026 Oct 6;2026(4):hoag089. doi: 10.1093/hropen/hoag089 (PMC13716481; doi:10.1093/hropen/hoag089)
Supplement: Supplementary file 1 [file hoag089_supplementary_data.docx]

**Supplementary figures and tables**

**Prenatal exposure to paracetamol is associated with smaller testicular volume: A COPANA cohort study of 287 healthy infant boys**

Gylli Mola^1,2^, Margit Bistrup Fischer^1,2^, Hanne Frederiksen^1,2^, Jørgen Holm Petersen^1^, Lone Scheel^3^, Katrine Bak Wraae^3^, Ane Lilleøre Rom^4,5^, Hanne Kristine Hegaard^4,6^, David Møbjerg Kristensen^1,7^, Anders Juul^1,2,6^, Casper P. Hagen^1,2,6^

^1^Department of Growth and Reproduction, Copenhagen University Hospital - Rigshospitalet, Copenhagen, Denmark

^2^International Centre for Research and Research Training in Endocrine Disruption of Male Reproduction and Child Health (EDMaRC), Copenhagen University Hospital - Rigshospitalet, Copenhagen, Denmark

^3^Center of Fetal Medicine and Pregnancy, Department of Obstetrics, Copenhagen University Hospital - Rigshospitalet, Copenhagen, Denmark

^4^Department of Gynaecology, Fertility and Obstetrics, Copenhagen University Hospital - Rigshospitalet, Copenhagen, Denmark

^5^Research Unit of Gynaecology and Obstetrics, Department of Clinical Research, University of Southern Denmark, Odense, Denmark

^6^Department of Clinical Medicine, University of Copenhagen, Copenhagen, Denmark

^7^Department of Science and Environment, Roskilde University, Denmark.

**Supplementary Fig S1**. Study timeline. Questionnaire 1 (Q1) captured current self-reported medication use, Questionnaire 2 (Q2) captured general and reproductive health, and Questionnaire 3 (Q3) consisted of electronic questionnaires sent biweekly throughout pregnancy, capturing all medication use. Gestational week (GW)

**Supplementary Fig S2.** Associations between cumulative prenatal paracetamol dose across pregnancy and primary reproductive outcomes.
Adjusted differences in testicular volume, fetal anogenital distance (AGD), postnatal anogenital distance from the anus to the scrotum (AGDas), and testosterone levels according to cumulative maternal paracetamol dose across pregnancy, irrespective of exposure timing. Prenatally unexposed boys served as the reference group, and exposed boys were divided into tertiles according to the distribution of cumulative dose among exposed participants. Estimates are presented with 95% confidence intervals. Models were adjusted for maternal smoking and alcohol use in early pregnancy, maternal pre-pregnancy BMI, maternal use of acetylsalicylic acid (ASA) and non-steroidal anti-inflammatory drugs (NSAIDs) during pregnancy, and postnatal paracetamol use by the child. The fetal AGD model was additionally adjusted for estimated fetal weight, and the postnatal AGDas model for body weight at examination. Filled triangles indicate associations of borderline statistical significance (0.05 < p ≤ 0.10), and open circles indicate non-significant associations (p > 0.10). Standard deviation score (SDS).

**Supplementary Table S1.** Hormone analyses in infant blood samples (n = 245)

| Hormone name | Abbreviation | No. of samples analysed | % ≥ LOD | LOD | Unit | Method used |
| --- | --- | --- | --- | --- | --- | --- |
| Anti-Müllerian hormone | AMH | 243 | 100 | 0.14 | pmol/L | Chemiluminescence  immunoassay |
| Follicle-stimulating hormone | FSH | 240 | 100 | 0.3 | IU/L | Chemiluminescence  immunoassay |
| Luteinizing hormone | LH | 241 | 100 | 0.07 | IU/L | Chemiluminescence  immunoassay |
| Inhibin B |  | 238 | 100 | 3 | ng/L | Enzyme-linked  immunosorbent assay |
| Sexual hormone binding globuline | SHBG | 245 | 100 | 0.33 | nmol/L | Chemiluminescence  immunoassay |
| Testosterone | T | 245 | 100 | 0.031 | nmol/L | LC-MS/MS |
| Dehydroepiandrosterone sulfate | DHEAS | 245 | 100 | 4.51 | nmol/L | LC-MS/MS |
| Androstenedione | Adione | 245 | 100 | 0.031 | nmol/L | LC-MS/MS |
| Dihydroxytestosterone | DHT | 245 | 100 | 0.12 | nmol/L | LC-MS/MS |
| Progesterone | PROG | 245 | 100 | 0.036 | nmol/L | LC-MS/MS |
| 17α-Hydroxypregnolone | 17-OHPreg | 245 | 100 | 0.193 | nmol/L | LC-MS/MS |
| 17α-Hydroxyprogesterone | 17-OHP | 245 | 100 | 0.033 | nmol/L | LC-MS/MS |
| 11-Deoxycorticosterone | DOC | 245 | 100 | 0.029 | nmol/L | LC-MS/MS |
| Corticosterone | CORT | 245 | 100 | 0.217 | nmol/L | LC-MS/MS |
| Aldosterone | ALDO | 245 | 100 | 0.038 | nmol/L | LC-MS/MS |
| 11-Deoxycortisol | 11-DOC | 245 | 100 | 0.042 | nmol/L | LC-MS/MS |
| Cortisone |  | 245 | 100 | 0.642 | nmol/L | LC-MS/MS |
| Cortisol |  | 245 | 100 | 5.565 | nmol/L | LC-MS/MS |

LC/MS-MS: liquid chromatography-tandem mass spectrometry, LOD: limit of detection.

**Supplementary Table S2.** Outcome measures presented as mean (SD) stratified by prenatal paracetamol exposure

|  | **n** | **Total** | **n** | **Unexposed** | **n** | **Exposure any time during pregnancy** | **n** | **Combined early and late exposure** |
| --- | --- | --- | --- | --- | --- | --- | --- | --- |
| **Primary outcomes** |  |  |  |  |  |  |  |  |
| Testicular volume (mm3) | 277 | 423.4 (101.6) | 147 | 435.7 (110.6) | 130 | 409.5 (88.9) | 60 | 401.5 (80.6) |
| Fetal AGD (mm) | 284 | 21.4 (3.6) | 150 | 21.5 (3.5) | 134 | 21.2 (3.6) | 60 | 21.0 (3.4) |
| AGDas (mm) | 287 | 32.0 (5.6) | 150 | 31.9 (6.0) | 137 | 32.2 (5.2) | 62 | 32.4 (4.5) |
| T (SDS) | 245 | 0.29 (0.87) | 127 | 0.29 (0.90) | 118 | 0.28 (0.85) | 52 | 0.30 (0.85) |
| **Secondary outcomes** |  |  |  |  |  |  |  |  |
| AGDap (mm) | 287 | 63.5 (7.7) | 150 | 63.7 (8.4) | 137 | 63.3 (6.9) | 62 | 63.9 (6.7) |
| Penile width (mm) | 287 | 13.4 (1.7) | 150 | 13.4 (1.7) | 137 | 13.3 (1.7) | 62 | 13.2 (1.7) |
| Penile length (mm) | 287 | 27.0 (4.3) | 150 | 26.9 (4.4) | 137 | 27.2 (4.3) | 62 | 27.5 (4.6) |
| LH (SDS) | 241 | -0.05 (0.72) | 126 | -0.04 (0.70) | 115 | -0.05 (0.75) | 51 | -0.03 (0.66) |
| FSH (SDS) | 240 | 0.17 (1.04) | 126 | 0.22 (0.95) | 114 | 0.10 (1.14) | 51 | 0.10 (0.97) |
| LH/FSH | 240 | 1.27 (0.76) | 126 | 1.22 (0.61) | 114 | 1.34 (0.89) | 51 | 1.40 (1.02) |
| LH/T | 241 | 0.53 (0.29) | 126 | 0.54 (0.31) | 115 | 0.52 (0.27) | 51 | 0.54 (0.29) |
| AMH (SDS) | 243 | -0.01 (0.97) | 127 | -0.09 (0.94) | 116 | 0.09 (1.00) | 52 | 0.10 (0.96) |
| Inhibin B (SDS) | 238 | -0.03 (0.94) | 125 | -0.03 (0.91) | 113 | -0.03 (0.97) | 52 | -0.10 (1.01) |
| SHBG (SDS) | 235 | -0.09 (0.98) | 124 | -0.04 (0.89) | 111 | -0.16 (1.08) | 52 | -0.08 (1.04) |
| PROG (SDS) | 245 | -0.57 (1.08) | 127 | -0.38 (1.04) | 118 | -0.77 (1.09) | 52 | -0.93 (1.01) |
| 17-OHPreg (SDS) | 245 | -0.03 (1.12) | 127 | 0.12 (1.17) | 118 | -0.20 (1.05) | 52 | -0.30 (0.91) |
| 17-OHP (SDS) | 245 | 0.14 (0.91) | 127 | 0.31 (0.90) | 118 | -0.04 (0.90) | 52 | -0.11 (0.87) |
| Free T (pmol/L) | 235 | 26.66 (10.20) | 124 | 26.83 (10.68) | 111 | 26.47 (9.69) | 52 | 25.73 (9.60) |
| DHEAS (SDS) | 245 | -0.61 (1.40) | 127 | -0.50 (1.25) | 118 | -0.72 (1.54) | 52 | -0.69 (1.50) |
| Adione (SDS) | 245 | -0.24 (1.10) | 127 | -0.11 (1.00) | 118 | -0.38 (1.19) | 52 | -0.38 (1.15) |
| DHT (SDS) | 245 | 0.12 (0.74) | 127 | 0.09 (0.74) | 118 | 0.14 (0.73) | 52 | 0.15 (0.78) |
| DOC (SDS) | 245 | 0.14 (1.01) | 127 | 0.20 (1.04) | 118 | 0.06 (0.96) | 52 | 0.09 (0.92) |
| CORT (SDS) | 245 | 0.10 (0.98) | 127 | 0.16 (1.01) | 118 | 0.05 (0.94) | 52 | 0.08 (0.78) |
| ALDO (SDS) | 245 | 0.24 (0.80) | 127 | 0.27 (0.80) | 118 | 0.20 (0.81) | 52 | 0.23 (0.77) |
| 11-DOC (SDS) | 245 | 0.09 (1.09) | 127 | 0.20 (1.08) | 118 | -0.04 (1.09) | 52 | -0.06 (1.15) |
| Cortisol (SDS) | 245 | 0.22 (0.89) | 127 | 0.27 (0.92) | 118 | 0.17 (0.85) | 52 | 0.16 (0.76) |
| Cortisone (SDS) | 245 | 0.17 (0.92) | 127 | 0.21 (0.96) | 118 | 0.13 (0.88) | 52 | 0.14 (0.88) |

11-deoxycorticosterone (DOC), 11-deoxycortisol (11-DOC), 17-hydroxypregnenolone (17-OHPreg), 17-hydroxyprogesterone (17-OHP), aldosterone (ALDO), androstenedione (Adione), anti-müllerian hormone (AMH), ano-genital distance (AGD), ano-scrotal anogenital distance (AGDas), ano-penile anogenital distance (AGDap), corticosterone (CORT), dehydroepiandrosterone sulfate (DHEAS), dihydrotestosterone (DHT), progesterone (PROG), sex hormone binding globulin (SHBG), standard deviation score (SDS), testosterone (T).

**Supplementary Table S3.** Multiple regression analyses examining early and late fetal paracetamol exposure and their interaction in relation to predefined outcomes.

|  | Estimate (95% CI) | p value |
| --- | --- | --- |
| **Primary outcomes** |  |  |
| Testicular volume (mm³) (n=269) | | |
| Paracetamol exposure in early fetal life | -35.9 (-86.2; 14.4) | 0.161 |
| Paracetamol exposure in late fetal life | -14.8 (-47.3; 17.8) | 0.373 |
| Interaction term: early × late exposure | 18.5 (-44.7; 81.7) | 0.565 |
| Fetal AGD (mm) (n=276) | | |
| Paracetamol exposure in early fetal life | 0.26 (-1.25; 1.76) | 0.735 |
| Paracetamol exposure in late fetal life | 0.02 (-0.93; 0.97) | 0.967 |
| Interaction term: early × late exposure | -1.26 (-3.14; 0.62) | 0.187 |
| AGDas (mm) (n=279) | | |
| Paracetamol exposure in early fetal life | -0.82 (-3.51; 1.87) | 0.551 |
| Paracetamol exposure in late fetal life | 0.15 (-1.53; 1.83) | 0.862 |
| Interaction term: early × late exposure | 0.83 (-2.51; 4.17) | 0.626 |
| T (SDS) (n=237) | | |
| Paracetamol exposure in early fetal life | -0.01 (-0.48; 0.45) | 0.951 |
| Paracetamol exposure in late fetal life | -0.01 (-0.30; 0.29) | 0.965 |
| Interaction term: early × late exposure | 0.10 (-0.48; 0.68) | 0.738 |
| **Secondary outcomes** |  |  |
| AGDap (mm) (n=279) | | |
| Paracetamol exposure in early fetal life | -2.38 (-5.77; 1.01) | 0.168 |
| Paracetamol exposure in late fetal life | -0.33 (-2.44; 1.79) | 0.761 |
| Interaction term: early × late exposure | 2.76 (-1.46; 6.97) | 0.199 |
| Penile width (mm) (n=279) | | |
| Paracetamol exposure in early fetal life | -0.23 (-1.06; 0.59) | 0.579 |
| Paracetamol exposure in late fetal life | -0.10 (-0.62; 0.41) | 0.694 |
| Interaction term: early × late exposure | 0.05 (-0.97; 1.08) | 0.916 |
| Penile length (mm) (n=279) | | |
| Paracetamol exposure in early fetal life | 0.15 (-2.03; 2.33) | 0.891 |
| Paracetamol exposure in late fetal life | 0.03 (-1.33; 1.39) | 0.963 |
| Interaction term: early × late exposure | 0.68 (-2.02; 3.39) | 0.619 |
| LH (SDS) (n=233) | | |
| Paracetamol exposure in early fetal life | 0.10 (-0.29; 0.50) | 0.606 |
| Paracetamol exposure in late fetal life | -0.09 (-0.35; 0.16) | 0.470 |
| Interaction term: early × late exposure | 0.04 (-0.46; 0.54) | 0.878 |
| FSH (SDS) (n=232) | | |
| Paracetamol exposure in early fetal life | 0.01 (-0.55; 0.57) | 0.972 |
| Paracetamol exposure in late fetal life | -0.11 (-0.46; 0.25) | 0.564 |
| Interaction term: early × late exposure | -0.04 (-0.74; 0.67) | 0.918 |
| LH/FSH ratio (n=232) | | |
| Paracetamol exposure in early fetal life | 0.03 (-0.36; 0.42) | 0.875 |
| Paracetamol exposure in late fetal life | 0.00 (-0.25; 0.26) | 0.974 |
| Interaction term: early × late exposure | 0.17 (-0.33; 0.66) | 0.501 |
| LH/T ratio (n=233) | | |
| Paracetamol exposure in early fetal life | 0.01 (-0.14; 0.17) | 0.855 |
| Paracetamol exposure in late fetal life | -0.06 (-0.16; 0.04) | 0.229 |
| Interaction term: early × late exposure | 0.04 (-0.16; 0.23) | 0.723 |
| AMH (SDS) (n=235) | | |
| Paracetamol exposure in early fetal life | -0.15 (-0.65; 0.36) | 0.569 |
| Paracetamol exposure in late fetal life | 0.13 (-0.19; 0.45) | 0.435 |
| Interaction term: early × late exposure | 0.17 (-0.47; 0.80) | 0.601 |
| Inhibin B (SDS) (n=230) | | |
| Paracetamol exposure in early fetal life | -0.45 (-0.95; 0.05) | 0.076 |
| Paracetamol exposure in late fetal life | 0.17 (-0.16; 0.50) | 0.307 |
| Interaction term: early × late exposure | 0.21 (-0.42; 0.84) | 0.511 |
| SHBG (SDS) (n=227) | | |
| Paracetamol exposure in early fetal life | 0.18 (-0.38; 0.74) | 0.528 |
| Paracetamol exposure in late fetal life | -0.23 (-0.58; 0.11) | 0.183 |
| Interaction term: early × late exposure | 0.06 (-0.63; 0.75) | 0.865 |
| PROG (SDS) (n=237) | | |
| Paracetamol exposure in early fetal life | 0.08 (-0.50; 0.65) | 0.793 |
| Paracetamol exposure in late fetal life | -0.36 (-0.72; -0.00) | 0.048 |
| Interaction term: early × late exposure | -0.22 (-0.94; 0.49) | 0.541 |
| 17-OHPreg (SDS) (n=237) | | |
| Paracetamol exposure in early fetal life | -0.18 (-0.79; 0.42) | 0.549 |
| Paracetamol exposure in late fetal life | -0.27 (-0.64; 0.11) | 0.170 |
| Interaction term: early × late exposure | 0.02 (-0.74; 0.77) | 0.965 |
| 17-OHP (SDS) (n=237) | | |
| Paracetamol exposure in early fetal life | -0.26 (-0.74; 0.23) | 0.298 |
| Paracetamol exposure in late fetal life | -0.34 (-0.64; -0.04) | 0.029 |
| Interaction term: early × late exposure | 0.22 (-0.39; 0.82) | 0.482 |
| Free T (pmol/L) (n=227) | | |
| Paracetamol exposure in early fetal life | -1.50 (-7.38; 4.38) | 0.615 |
| Paracetamol exposure in late fetal life | 0.91 (-2.69; 4.52) | 0.618 |
| Interaction term: early × late exposure | 0.12 (-7.13; 7.37) | 0.974 |
| DHEAS (SDS) (n=237) | | |
| Paracetamol exposure in early fetal life | -0.44 (-1.18; 0.31) | 0.248 |
| Paracetamol exposure in late fetal life | -0.18 (-0.64; 0.29) | 0.461 |
| Interaction term: early × late exposure | 0.24 (-0.68; 1.17) | 0.604 |
| Adione (SDS) (n=237) | | |
| Paracetamol exposure in early fetal life | -0.35 (-0.94; 0.24) | 0.244 |
| Paracetamol exposure in late fetal life | -0.27 (-0.64; 0.11) | 0.161 |
| Interaction term: early × late exposure | 0.30 (-0.44; 1.04) | 0.429 |
| DHT (SDS) (n=237) | | |
| Paracetamol exposure in early fetal life | 0.15 (-0.24; 0.54) | 0.446 |
| Paracetamol exposure in late fetal life | 0.03 (-0.22; 0.27) | 0.834 |
| Interaction term: early × late exposure | -0.06 (-0.55; 0.42) | 0.798 |
| DOC (SDS) (n=237) | | |
| Paracetamol exposure in early fetal life | -0.09 (-0.61; 0.43) | 0.733 |
| Paracetamol exposure in late fetal life | -0.25 (-0.58; 0.08) | 0.135 |
| Interaction term: early × late exposure | 0.27 (-0.38; 0.93) | 0.410 |
| CORT (SDS) (n=237) | | |
| Paracetamol exposure in early fetal life | -0.13 (-0.65; 0.38) | 0.609 |
| Paracetamol exposure in late fetal life | -0.22 (-0.55; 0.10) | 0.178 |
| Interaction term: early × late exposure | 0.29 (-0.36; 0.94) | 0.384 |
| Aldo (SDS) (n=237) | | |
| Paracetamol exposure in early fetal life | -0.07 (-0.51; 0.36) | 0.741 |
| Paracetamol exposure in late fetal life | -0.11 (-0.38; 0.17) | 0.440 |
| Interaction term: early × late exposure | 0.21 (-0.34; 0.75) | 0.453 |
| 11-DOC (SDS) (n=237) | | |
| Paracetamol exposure in early fetal life | -0.34 (-0.92; 0.23) | 0.243 |
| Paracetamol exposure in late fetal life | -0.28 (-0.64; 0.08) | 0.126 |
| Interaction term: early × late exposure | 0.34 (-0.38; 1.06) | 0.355 |
| Cortisol (SDS) (n=237) | | |
| Paracetamol exposure in early fetal life | -0.10 (-0.57; 0.38) | 0.686 |
| Paracetamol exposure in late fetal life | -0.15 (-0.44; 0.15) | 0.333 |
| Interaction term: early × late exposure | 0.08 (-0.52; 0.67) | 0.801 |
| Cortisone (SDS) (n=237) | | |
| Paracetamol exposure in early fetal life | -0.42 (-0.92; 0.08) | 0.100 |
| Paracetamol exposure in late fetal life | -0.00 (-0.32; 0.31) | 0.995 |
| Interaction term: early × late exposure | 0.32 (-0.31; 0.95) | 0.313 |

Data are presented as β coefficients with 95% confidence intervals. 11-deoxycorticosterone (DOC), 11-deoxycortisol (11-DOC), 17-hydroxypregnenolone (17-OHPreg), 17-hydroxyprogesterone (17-OHP), aldosterone (ALDO), androstenedione (Adione), anti-müllerian hormone (AMH), ano-genital distance (AGD), ano-scrotal anogenital distance (AGDas), ano-penile anogenital distance (AGDap), corticosterone (CORT), dehydroepiandrosterone sulfate (DHEAS), dihydrotestosterone (DHT), progesterone (PROG), sex hormone binding globulin (SHBG), standard deviation score (SDS), testosterone (T). Adjusted models include pre-pregnancy BMI, maternal nicotine and alcohol use in the first trimester of pregnancy, maternal acetylsalicylic acid (ASA) and non-steroidal anti-inflammatory drugs (NSAIDs) use during pregnancy, and child paracetamol use. Analyses of fetal AGD were additionally body parameter adjusted for estimated fetal weight, and analyses of AGDas and AGDap were additionally body parameter adjusted for body weight at examination. *indicates a p-value < 0.05.

**Supplementary Table S4.** Sensitivity analyses excluding women reporting fever as indication for paracetamol use (n=29), and women with maternal PCOS (n=11), respectively. Multiple linear regression analyses of reproductive and steroid hormone outcomes according to prenatal paracetamol exposure.

|  | **Early exposure** | | | | **Late exposure** | | | | **Combined early and late exposure** | | | |
| --- | --- | --- | --- | --- | --- | --- | --- | --- | --- | --- | --- | --- |
|  | Adjusted for late exposure | | Adjusted for additional covariates | | Adjusted for early exposure | | Adjusted for additional covariates | | Sum of early and late exposure§ | | Sum of early and late exposure adjusted for additional covariates | |
| **Excluding women reporting fever n=29** | | | | |  |  |  |  |  |  |  |  |
| **Outcome** | **n** |  | **n** |  | **n** |  | **n** |  | **n** |  | **n** |  |
| Testicular volume (mm³) | 62 | -19.5 (-52.8; 13.8) | 60 | -17.5 (-52.2; 17.1) | 84 | -18.3 (-48.7; 12.2) | 83 | -15.3 (-45.9; 15.4) | 44 | -37.8 (-71.2; -4.4)* | 43 | -32.8 (-67.9; 2.4) |
| PROG (SDS) | 55 | 0.02 (-0.34; 0.37) | 53 | 0.02 (-0.37; 0.41) | 77 | -0.46 (-0.78; -0.13)* | 76 | -0.44 (-0.77; -0.10)* | 39 | -0.44 (-0.80; -0.08)* | 38 | -0.42 (-0.82; -0.02)* |
| 17-OHPreg (SDS) | 55 | -0.13 (-0.51; 0.26) | 53 | -0.14 (-0.55; 0.28) | 77 | -0.28 (-0.63; 0.07) | 76 | -0.27 (-0.63; 0.09) | 39 | -0.41 (-0.80; -0.02)* | 38 | -0.41 (-0.83; 0.01) |
| 17-OHP (SDS) | 55 | -0.05 (-0.36; 0.25) | 53 | -0.05 (-0.38; 0.28) | 77 | -0.30 (-0.58; -0.02)* | 76 | -0.27 (-0.55; 0.02) | 39 | -0.35 (-0.66; -0.04)* | 38 | -0.32 (-0.65; 0.01) |
| DHEAS (SDS) | 55 | -0.24 (-0.72; 0.24) | 53 | -0.35 (-0.85; 0.15) | 77 | -0.10 (-0.54; 0.34) | 76 | -0.20 (-0.64; 0.24) | 39 | -0.34 (-0.83; 0.15) | 38 | -0.55 (-1.07; -0.04)* |
| Adione (SDS) | 55 | -0.11 (-0.48; 0.26) | 53 | -0.16 (-0.55; 0.24) | 77 | -0.22 (-0.56; 0.11) | 76 | -0.24 (-0.58; 0.10) | 39 | -0.34 (-0.71; 0.04) | 38 | -0.40 (-0.80; 0.01) |
| 11-DOC (SDS) | 55 | -0.05 (-0.43; 0.33) | 53 | -0.10 (-0.50; 0.29) | 77 | -0.18 (-0.52; 0.16) | 76 | -0.18 (-0.52; 0.17) | 39 | -0.23 (-0.61; 0.15) | 38 | -0.28 (-0.69; 0.13) |
| **Excluding women with PCOS n=11** | | | | |  |  |  |  |  |  |  |  |
| **Outcome** | **n** |  | **n** |  | **n** |  | n |  | n |  | n |  |
| Testicular volume (mm³) | 77 | -25.2 (-55.6; 5.3) | 74 | -26.7 (-58.2; 4.8) | 107 | -8.3 (-36.5; 19.8) | 105 | -6.1 (-34.6; 22.5) | 58 | -33.5 (-64.1; -3.0)* | 56 | -32.8 (-65.3; -0.3)* |
| PROG (SDS) | 66 | -0.10 (-0.44; 0.23) | 63 | -0.09 (-0.45; 0.26) | 98 | -0.45 (-0.75; -0.14)* | 96 | -0.43 (-0.75; -0.11)* | 49 | -0.55 (-0.90; -0.20)* | 47 | -0.52 (-0.90; -0.14)* |
| 17-OHPreg (SDS) | 66 | -0.18 (-0.53; 0.17) | 63 | -0.20 (-0.58; 0.17) | 98 | -0.27 (-0.59; 0.05) | 96 | -0.28 (-0.61; 0.05) | 49 | -0.45 (-0.81; -0.08)* | 47 | -0.48 (-0.88; -0.09)* |
| 17-OHP (SDS) | 66 | -0.14 (-0.42; 0.15) | 63 | -0.15 (-0.44; 0.15) | 98 | -0.31 (-0.57; -0.05)* | 96 | -0.30 (-0.56; -0.03)* | 49 | -0.45 (-0.74; -0.16)* | 47 | -0.44 (-0.76; -0.13)* |
| DHEAS (SDS) | 66 | -0.20 (-0.64; 0.24) | 63 | -0.23 (-0.69; 0.23) | 98 | -0.01 (-0.41; 0.39) | 96 | -0.08 (-0.49; 0.33) | 49 | -0.21 (-0.67; 0.25) | 47 | -0.31 (-0.80; 0.18) |
| Adione (SDS) | 66 | -0.13 (-0.48; 0.22) | 63 | -0.15 (-0.52; 0.22) | 98 | -0.17 (-0.49; 0.15) | 96 | -0.19 (-0.52; 0.14) | 49 | -0.31 (-0.67; 0.06) | 47 | -0.34 (-0.73; 0.05) |
| 11-DOC (SDS) | 66 | -0.09 (-0.43; 0.26) | 63 | -0.14 (-0.49; 0.22) | 98 | -0.18 (-0.49; 0.14) | 96 | -0.20 (-0.52; 0.12) | 49 | -0.26 (-0.62; 0.10) | 47 | -0.34 (-0.71; 0.04) |

Data are presented as β coefficients with 95% confidence intervals from unadjusted and adjusted linear regression models. Exposure was analysed in a multi-category model including early pregnancy exposure and late pregnancy exposure, with unexposed as the reference group. Combined early + late exposure represents the sum of the estimates for early and late exposure, where §early exposure is adjusted for late, and late exposure is adjusted for early. 11-deoxycortisol (11-DOC), 17-hydroxyprogesterone (17-OHP), 17-hydroxypregnenolone (17-OHPreg), androstenedione (Adione), dehydroepiandrosterone sulfate (DHEAS), progesterone (PROG), standard deviation score (SDS). Adjusted models include pre-pregnancy BMI, maternal nicotine and alcohol use in the first trimester of pregnancy, maternal ASA and NSAID use during pregnancy, and child paracetamol use. *indicates a p-value < 0.05.
